# Rapid Generation of Multiple Loci-Engineered Marker-free Poxvirus and Characterization of a Clinical-Grade Oncolytic Vaccinia Virus

**DOI:** 10.1016/j.omtm.2017.09.007

**Published:** 2017-09-30

**Authors:** Zong Sheng Guo, Zuqiang Liu, Magesh Sathaiah, Jiahu Wang, Roshni Ravindranathan, Eun Kim, Shaohua Huang, Thomas W. Kenniston, John C. Bell, Herbert J. Zeh, Lisa H. Butterfield, Andrea Gambotto, David L. Bartlett

**Affiliations:** 1 UPMC Hillman Cancer Center and Department of Surgery, University of Pittsburgh School of Medicine, Pittsburgh, PA, USA; 2 Centre for Innovative Cancer Research, Ottawa Hospital Research Institute, Ottawa, ON K1H 8L6, Canada; 3 Departments of Medicine and Immunology, University of Pittsburgh School of Medicine, Pittsburgh, PA, USA

**Keywords:** vaccinia virus, oncolytic virus, vaccine, cancer, method, marker-free poxvirus, clinical grade, immunotherapy

## Abstract

Recombinant poxviruses, utilized as vaccine vectors and oncolytic viruses, often require manipulation at multiple genetic loci in the viral genome. It is essential for viral vectors to possess no adventitious mutations and no (antibiotic) selection marker in the final product for human patients in order to comply with the guidance from the regulatory agencies. Rintoul et al. have previously developed a selectable and excisable marker (SEM) system for the rapid generation of recombinant vaccinia virus. In the current study, we describe an improved methodology for rapid creation and selection of recombinant poxviruses with multiple genetic manipulations solely based on expression of a fluorescent protein and with no requirement for drug selection that can lead to cellular stress and the risk of adventitious mutations throughout the viral genome. Using this improved procedure combined with the SEM system, we have constructed multiple marker-free oncolytic poxviruses expressing different cytokines and other therapeutic genes. The high fidelity of inserted DNA sequences validates the utility of this improved procedure for generation of therapeutic viruses for human patients. We have created an oncolytic poxvirus expressing human chemokine CCL5, designated as vvDD-A34R-hCCL5, with manipulations at two genetic loci in a single virus. Finally, we have produced and purified this virus in clinical grade for its use in a phase I clinical trial and presented data on initial in vitro characterization of the virus.

## Introduction

Poxviruses are a large family of DNA viruses that can infect a wide range of hosts. A well-studied poxvirus, vaccinia virus (VV) is the prototypic member of the *Orthopoxvirus* genus. The poxvirus genome, about 190 kb in size, can be modified genetically and can accommodate inserts of DNA fragments exceeding 25 kb in size without losing infectivity and other functions. Various strains of VV have been used as vaccines for the eradication of smallpox in the world. Since then, recombinant VV and other poxviruses have proven to be valuable vectors for protein expression, gene therapy imaging, and diagnosis.^1^, ^2^, ^3^, ^4^, ^5^ VV have been explored as vaccine vectors to express immunogens from infectious diseases such as acquired immunodeficiency syndrome,^6^ malaria,^7^ and tuberculosis^8^ and as vectors for cancer vaccines and immunotherapy.^9^, ^10^, ^11^, ^12^ More recently, VV and other poxviruses have been developed as oncolytic viruses for cancer therapy.^4^, ^12^, ^13^ We have shown that an oncolytic virus expressing chemokine CCL5 enhanced its therapeutic effects when compared to the parental virus vvDD,^14^ and an oncolytic virus (OV) together with an immune checkpoint blockade anti-PD-L1 antibody worked synergistically to enhance the therapeutic efficacy in cancer models.^15^ At least three strains of genetically engineered oncolytic VV have been evaluated in human cancer patients, and results have been published: Wyeth strain (Pexa-Vec),^16^ WR strain (vvDD-CDSR),^17^, ^18^ and Lister strain.^19^

High degrees of safety and potency are two critical features of a successful therapeutic drug for human patients. To achieve these goals in recombinant viruses, investigators have often genetically modified therapeutic viruses at multiple genetic loci. Typically, new poxviruses are generated by constructing a shuttle plasmid containing an expression cassette with the gene(s) of interest flanked by DNA sequences homologous to the desired target locus (such as the *tk* locus), followed by transfection of the plasmid into mammalian cells infected with a specific parental poxvirus to allow for recombination of the homologous sequences between the plasmid and the viral genomic DNA.^20^ Decades of research with wild-type and recombinant viruses including poxviruses led us to some valuable lessons. First, the frequency of recombination is typically less than 0.01%,^21^ and the isolation of recombinants is tedious and time consuming. Second, drugs are the most commonly used agents for selection of new recombinant viruses. For example, drugs have been used to select for *tk*-positive or -negative phenotypes,^20^ and resistances to mycophenolic acid^22^ or neomycin^23^ are often used to select new recombinant *tk*-mutated poxviruses. Third, despite relatively low mutation rates associated with the DNA viruses,^24^ recent studies have demonstrated that poxviruses evolve rapidly to adapt against host defense and become drug resistant with gene amplification and adaptive mutations in viral genes such as K3L and A17L.^25^, ^26^, ^27^, ^28^ It is well known that mutation is induced through multiple mechanisms as a response to cell stress in organisms from virus to man.^29^, ^30^, ^31^ Mammalian cells under stress can inhibit DNA mismatch repair, generating genotoxic stress, and thus can increase the mutation rates of both viral and host genomes.^29^, ^32^, ^33^, ^34^ These studies strongly suggest that mutations throughout the viral genome may happen during the selection process using antibiotics or other agents promoting high cellular stress and during amplification of the virus. In fact, Dambach et al.^35^ have reported the first documentation that oncolytic herpes viruses developed and used in clinical trials contained adventitious mutations. The authors have suggested that the use of bromodeoxyuridine in the media during selection for the γ34.5- and *tk*-deficient virus R3612 might be one of the most likely events leading to the G-C to A-T transition (mutation) taking place in the UL3 gene.

To minimize the mutation rate, methods that reduce cell stress are highly preferred for construction of new viruses intended for clinical use, especially for viruses with a large DNA genome such as poxviruses and herpes simplex virus. Fortunately, methods with lower cellular stress do exist. These methods include plaque purification and visual screening to purify new viruses expressing β-galactosidase^36^ or β-glucuronidase,^37^ which convert substrate compounds into color products, or expressing a fluorescent protein as reporter identifiable by naked eyes, microscopy, or flow cytometry.^38^

Two classes of methods have been frequently used to construct and isolate new recombinant poxvirus clones. The first one depends on the inclusion of drug resistance and/or reporter genes.^21^, ^23^ The second one is termed dominant host-range selection.^39^, ^40^, ^41^ Previously, Di Lullo et al.^21^ developed a new methodology to create recombinant VVs efficiently by using marker gene swapping and fluorescence-activated cell sorting. This has been one of the key developments leading to the current methodology of creating new poxviruses to be discussed in the current study. Later, Rintoul et al.^42^ further developed an efficient protocol to generate new poxviruses using double selection of drug (mycophenolic acid) and yellow fluorescent protein (YFP), via the selectable and excisable marker (SEM) system, that was based on the LoxP and Cre recombinase system of bacteriophage P1.^43^ In this report, we describe an improved low-cell-stress methodology to generate recombinant poxviruses with YFP as the only marker used for selection. The YFP (gene) marker in the virus could be then removed in the Cre-expressing cells. We have applied this newly improved procedure aimed to reduce potential mutation in viral genomes and have generated different versions of VV expressing human or murine chemokine CCL5 (RANTES), or cytokines such as IL-2, IL-12, IL-15, IL-23, GM-CSF, and the anti-PD-1 antibody Nivolumab, with genetic manipulations of up to three viral loci: *tk*, *vgf*, and A34R.^44^, ^45^ Here, we use the construction of vvDD-A34R-hCCL5, a marker-free and chemokine CCL5-expressing recombinant virus, as the first example for the improved procedure of construction and selection. Finally, we have produced a batch of this virus under manufacturing conditions compatible for its use in a phase I clinical trial, and present here data of the initial in vitro characterization of the clinical grade virus.

## Results

### Construction of Multiple Shuttle Vectors for Various Loci in the Viral Genome

**Figure 1:**
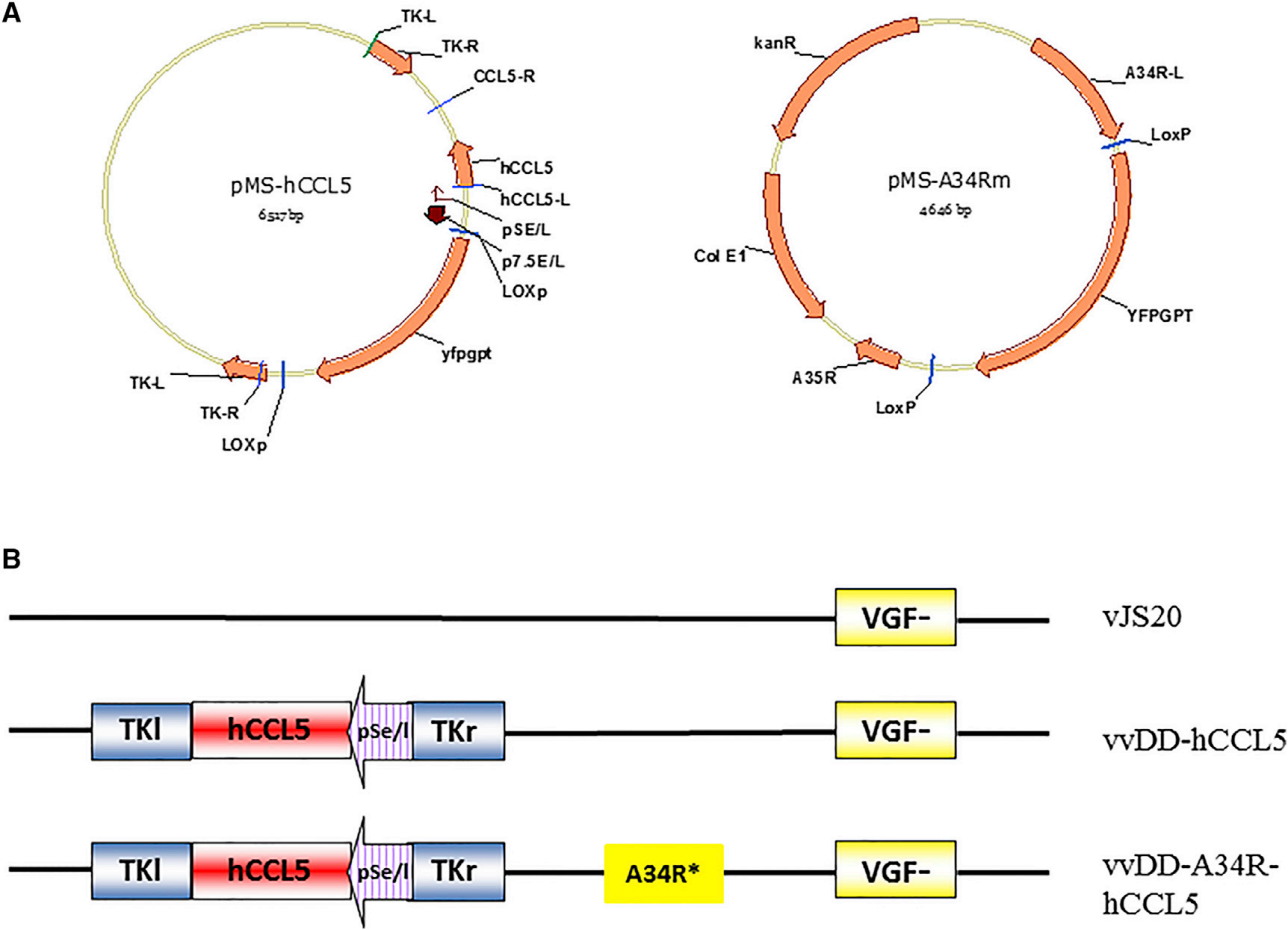
Schematic Representation of Shuttle Plasmids and Vaccinia Viruses Used or Constructed in This Study (A) Shuttle plasmids for the *tk* locus containing an expression cassette with human CCL5 cDNA (pMS-hCCL5) and for mutation in the A34R gene with (lys-151 changed to glu in the protein) (pMS-A34Rm). (B) Cartoons of parental and one intermediate viruses and final product vvDD-A34R-hCCL5.

Because of the need to manipulate multiple genetic loci in the viral genome, we have made shuttle vectors for the two loci: *tk* and *A34R*, which were constructed based on the original shuttle vector for the *tk* locus built previously, pSEM-1.^42^ This original vector expresses the fusion gene yfp-gpt, which allows the “double selection”: resistance to the drug mycophenolic acid and expression of YFP. The expression cassette was flanked by two loxP sites, thus allowing the removal of the enclosed DNA sequence in mammalian cells expressing the Cre recombinase. The shuttle vector for A34R locus was designed to introduce a mutation that results in more progeny virus in an extracellular enveloped virus (EEV) form. This A34R shuttle vector was constructed with a left-flanking sequence derived from the *A34R* gene with the specific point mutation (lysine-151 to glu) and a right-flanking sequence from *A35R* gene. The shuttle vectors for the *tk* locus carrying hCCL5 gene (pMS-hCCL5) and the A34R locus carrying the mutant A34R gene (pMS-A34R) are shown in Figure 1A. For the construction of vvDD-A34R-hCCL5, a number of intermediate viral vectors were generated, but only the parental virus vSC20, one of the intermediate viruses, vvDD-hCCL5, and the final product vvDD-A34R-hCCL5 are shown (Figure 1B).

### The Improved Procedure with No Use of Drug Selection

**Figure 2:**
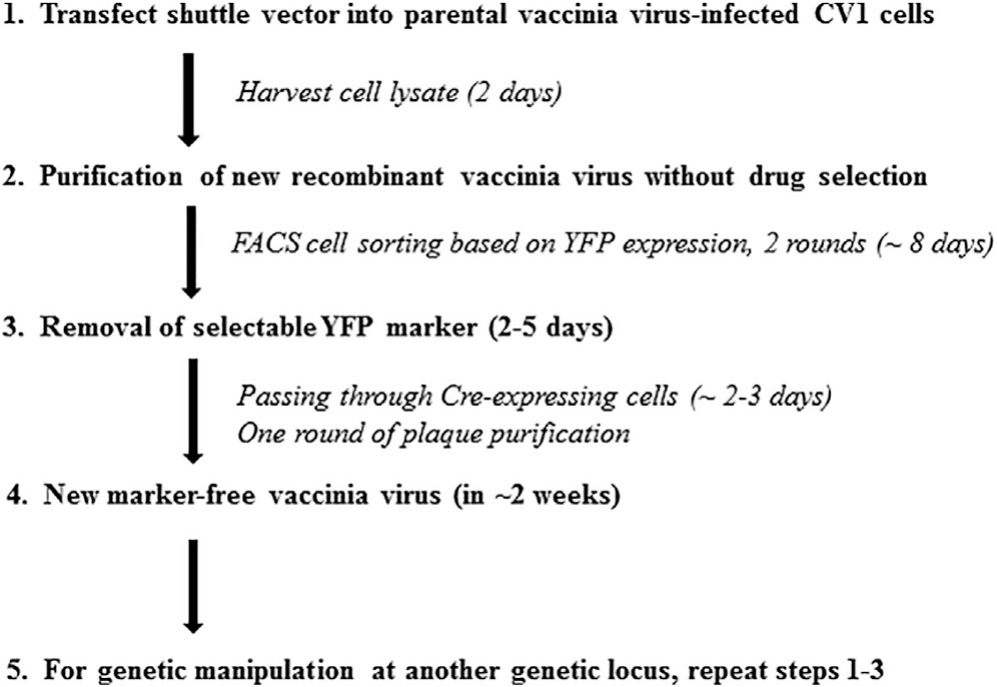
Schematic Presentation of the Improved Procedure for Recombination and Selection of New Recombinant VVs under Low Cell Stress

The improved procedure used to generate vvDD-A34R-hCCL5 is described in Figure 2. We have also used this improved procedure to construct vvDD-A34R-mCCL5, a related virus that expresses murine CCL5 instead of human CCL5. In addition, we have made versions of VVs expressing cytokines such as IL-2, IL-12, IL-15, IL-23, GM-CSF and the anti PD-1 antibody Nivolumab, using this improved procedure. Here we describe the improved procedure for construction of new recombinant poxviruses in greater details, using vvDD-A34R-hCCL5 as the example.

### Construction of vvDD-hCCL5-YFP in the Backbone of *tk*/*vgf* Dual Mutations

Below, we describe the construction of the virus expressing hCCL5 and YFP in the *tk* locus in the backbone of *vgf* mutation, thus resulting in a “double deleted virus” (vvDD), as we have called those VVs with *tk* and *vgf* gene mutations.^44^ As described in the Materials and Methods and Figure 2, CV-1 cells were infected with parental virus vSC20 and then transfected with plasmid pMS-hCCL5. Two days later, we observed about 15%–30% YPF-positive cells (mostly expression from the plasmid). The infected cells and supernatants were harvested, and the lysates were used to infect new CV-1 cells. All cells (one well in a 6-well plate) were harvested around 24 hr later and subjected to flow sorting, using uninfected CV-1 cells and cells infected with a well-characterized YFP-containing VV as negative and positive controls for gating purposes. The YFP-positive cells were sorted as single cells into the wells of a 96-well plate that was previously plated with uninfected CV-1 cells, at one cell per well. We usually observed about 0.1%–0.5% YPF-positive cells in the flow sorting at this stage. Depending on the quality of original transfection, we have routinely detected about 20% positive wells in the 96-well plate, with ∼80% wells as negative for YFP. After 3–5 days, we picked two to four wells with brightest YFP expression and harvested all of the cells in the wells as independent clones. The cell lysates were used to infect new CV-1 cells in 12-well plates. About 24 hr later, cells were harvested and subjected to flow sorting again, and YFP-positive cells were sorted into new CV-1 cells in 96-well plate, one cell per well. In these 96-well plates, we have observed up to ∼70% of wells expressing YFP. Again, two to four wells were picked as candidates for new recombinant virus vvDD-hCCL5-YFP. For some clones, we would do one to two rounds of plaque purification.

### Removal of YFP-GPT Marker Genes from the Virus in U2OS-Cre Cells

**Figure 3:**
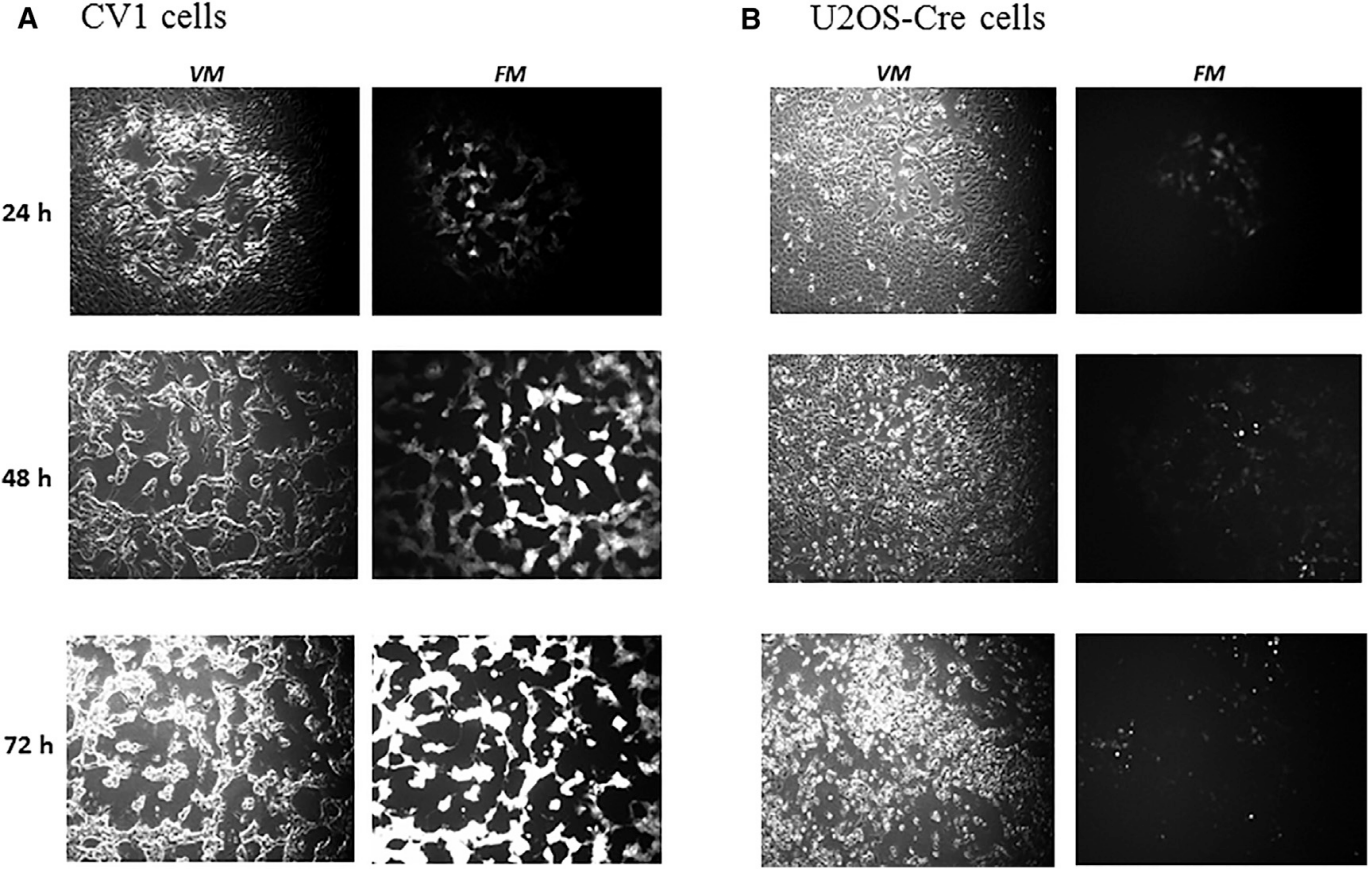
Generation of a Marker-free VV (vvDD-hCCL5) in U2OS-Cre Cells Both CV-1 (A) cells and U2OS (B) cells were plated at 2.0e5 cells per well in 6-well plates overnight and then infected with series dilution of the virus (1:3). Both visible light microscopy (*VM*) and fluorescence microscopy (*FM*) was used to observe the development of plaques and the expression of YFP as an indicator of the removal of marker genes, at 24, 48, and 72 hr. Pictures were taken on approximately same positions of cells at three time points. All pictures were taken with same amplification (10×).

To remove YFP-GPT marker genes from the virus, U2OS-Cre cells, and CV-1 cells (as negative control), in 6-well plates were infected with vvDD-hCCL5-YFP at low MOI (at 3-fold series dilutions). The infection and expression of YFP marker were monitored by microscopy (Figure 3). In CV-1 cells in which no Cre recombinase exists, we observed small plaques forming at 24 hr, with infected cells in the center of the plaque expressing YFP. By 48 hr after infection, larger plaque was formed, and many infected cells expressed highly bright fluorescence, indicating high levels of YFP. By 72 hr, essentially all cells in the field were infected with the virus and expressing large quantity of YFP protein (Figure 3A). In contrast, in U2OS-Cre cells, plaques started to form at 24 hr, increased in size over time, just like those in CV-1 cells (48 and 72 hr). However, the fluorescence was dim or not visible in cells infected with the virus, at all time (24, 48, and 72 hr) (Figure 3B). These results showed that Cre recombinase in the U2OS-Cre cells is expressed and able to remove the YPF marker gene-containing DNA fragment flanked by the two loxP sites in most progeny viruses.

Two to three days after infection, U2OS-Cre cells were harvested, cell lysates were made, and the series diluents were used to infect CV-1 cells in 96-well plate to isolate pure, YFP-negative plaques. A few clones were selected, and one was used to amplify. We designated the new virus without *yfp-gpt* marker genes as vvDD-hCCL5. The identities of the virus isolates were confirmed by gene-specific PCR for hCCL5 and YFP.

### Isolation of Viruses with Mutated A34R, with or without YFP Marker Gene

To introduce the A34R mutation into the virus, we infected CV-1 cells in one well of a 6-well plate with vvDD-hCCL5 at a low MOI (∼0.1) and then transfected the cells with the shuttle vector pMS-A34Rm (with mutant A34R gene and YFP gene in the shuttle vector). The two-round selection procedure (for YFP expression) was the same as descried above for the insertion of hCCL5-YFP into the *tk* locus (Figure 2). Through the procedure, new virus vvDD-A34R-YFP-hCCL5 was obtained. This YFP-expressing virus may be useful for preclinical studies, where YFP can be used to monitor the infection efficiency or as a marker for other purposes.

To obtain the final version of the virus lacking the YFP marker gene at the A34R locus, vvDD-A34R-YFP-hCCL5 was passed in U2OS-Cre cells, using the same procedure as described. One round of plaque purification was used to remove any residual YFP-positive virus.

### DNA Sequencing Confirmed the High Fidelity of the Inserted DNA Fragments

**Figure 4:**
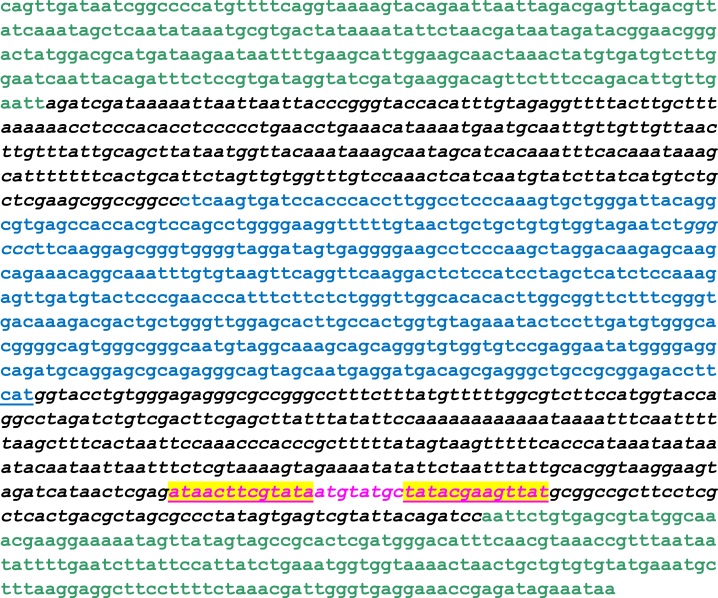
DNA Sequence Covering the Transgene Insert and Flanking *tk* Gene Sequence in the Viral Genome of vvDD-A34R-hCCL5 Viral genomic DNA was purified and the DNA fragment of 1.7 kb covering the transgene insert and flanking *tk* gene sequence was sequenced using 10 sequencing primers annealing in both orientations. DNA analytic software was used to align DNA sequences between those from the shuttle vector, databases, and sequencing reactions. The 1,591-bp fragment is presented here. It covers the 260-bp left-/204-bp right-flanking *tk* gene sequences (in bold green), 268-bp vector sequence (solid italic black), then ∼500-bp human CCL5 cDNA sequence in reverse orientation (in bold blue), 360-bp vector sequence containing the viral p7.5 E/L and pSE/L promoters in opposite orientation (in solid italic black). Inside this segment, the sequence *ataacttcgtataatgtatgctatacgaagttat* (highlighted yellow) indicates a *loxP* recognition site.

To confirm the high fidelity of the viral genome sequence of the viruses generated through this low cellular stress procedure, we isolated multiple independent viral plaques and four independent clones have undergone virus amplification, and their inserted DNA at the *tk* locus and the point mutation of A34R gene was confirmed by DNA sequencing. We have also sequenced other new viruses constructed through this protocol and found no mutations. In Figure 4, we presented the DNA sequence at the *tk* locus in the final virus product. Analysis confirmed the desirable insertion of wild-type human CCL5 cDNA sequence and viral *tk* gene sequence flanking the insertion (Figure 4).

### Production and Characterization of Clinical-Grade vvDD-A34R-hCCL5 In Vitro

In preparation for the planned phase I clinical trial with vvDD-A34R-hCCL5, we have produced and purified the virus in a clinical-grade setting in the GMP suite of the UPCI Cellular Products Laboratory (CPL). The cell line used, the procedures followed, and the GMP facility meet the requirements for production of a biological product for phase I clinical trial testing in human patients. We have used certified HEK293 cells to amplify the virus.

After expansion, purification, and viling, we performed a number of quality control assays and characterizations of the virus. These included safety testing, including endotoxin level and 14-day sterility testing. Also tested were the virus titer (in plaque-forming units [PFU]), electron microscopy of the virus preparation with or without sonication, viral replication and cytotoxicity in cancer cells, and production and secretion of human CCL5 from the infected cells into the medium. The virus was also subjected to toxicity study in mice.

Assays showed that the virus preparation was sterile, and mycoplasma negative (Table 1). Endotoxin content was <0.0500 EU/mL, while the buffer control was also <0.0500 EU/mL (below limit of detection). In addition, the titer of the virus, when stored in a −80°C freezer monitored in the CPL facility, was stable for at least l8 months. We have tested adventitious agents through contracted services (Wuxi AppTec, Philadelphia, PA). The results showed that an aliquot of the virus preparation was negative for 10 different viruses, including AAV, CMV, EBV, HBV, HCV, HIV-1, HIV-2, HILV-1, HTLV-2, and parvovirus B-19 (data not shown).

**Table 1:** Some Test Results on the Clinical-Grade vvDD-A34R-hCCL5

Test	Results
Physical appearance	clear, colorless liquid free from visible particulates
14-day sterility	sterile
Mycoplasma (by PCR assay)	negative
Endotoxin	0.0500 EU/mL (below the level of detection)
Titer of the virus in PFU (on CV-1 cells)	1.1e9 PFU/mL
Virus-particles-to-PFU ratio (based on optical density, OD_260_ = 1.95)	∼25:1
CCL5 production from infected HEK293 cells (MOI = 1.0)	∼600 ng/1.0e6 cells/48 hr

Note: Most tests were performed by contracted FDA-certified facilities.

**Figure 5:**
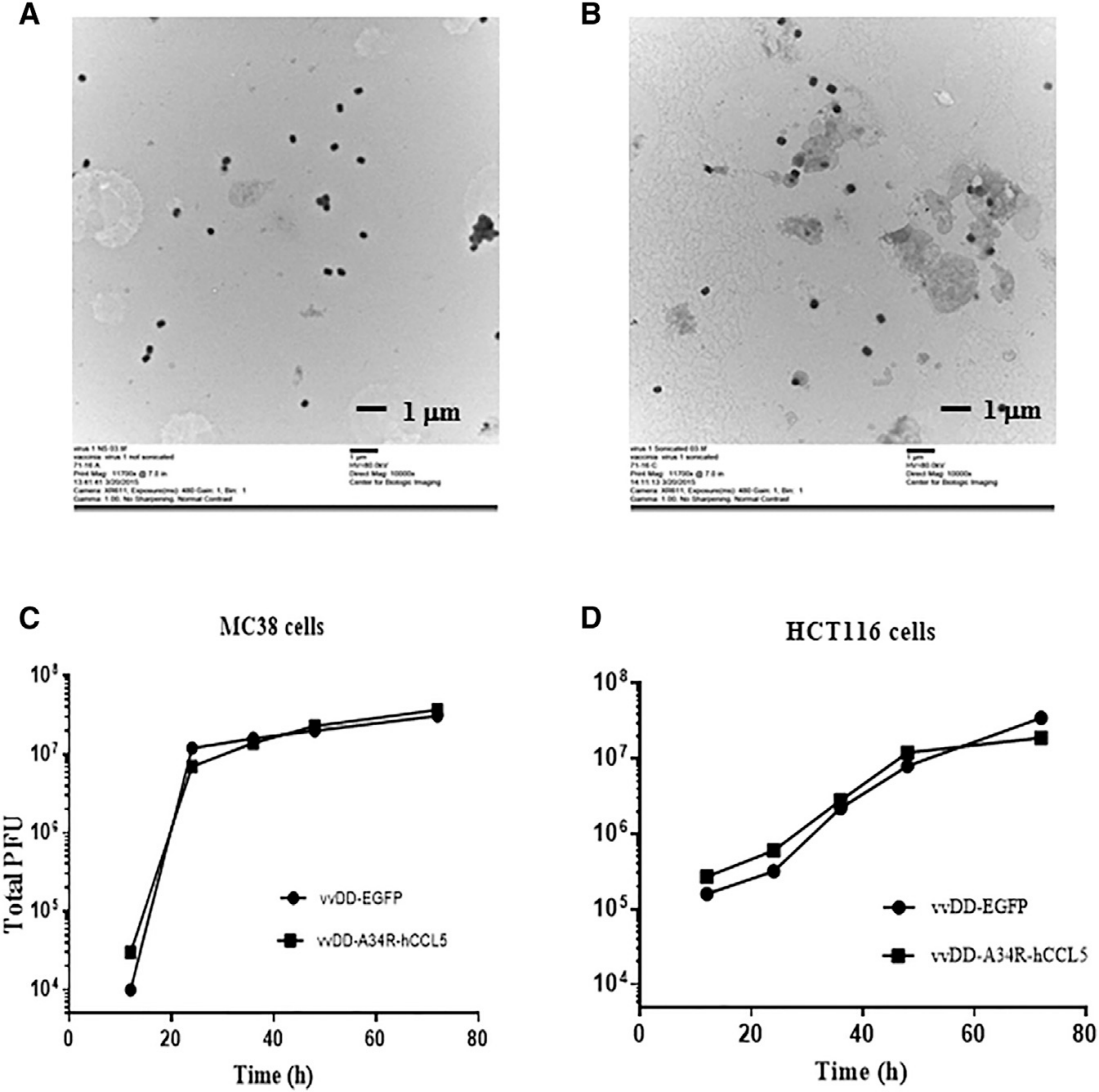
Preliminary Characterization of Clinical-Grade vvDD-A34R-hCCL5 In Vitro Shown are representative pictures of negative stain by electron microscopy of the poxvirus particles without (A) or with sonication (B). Viral replication in murine MC38 and human HCT116 colorectal cancer cells are shown in (C) and (D). MC38 and HCT116 colorectal cancer cells were infected at MOI of 1.0, and cells were harvested at various time points (12, 24, 36, 48, and 72 h), and the viruses in the cell lysates were titered in CV-1 cells. Data from MC38 (C) and HCT116 cancer cells (D) are presented.

Previous studies suggested that VV virions, coated with membranes, tend to form aggregates, likely with cell debris and also other virions, thus reducing the effective infectivity and viral titers. Therefore, sonication immediately before the application of the virus has been used in research laboratories. We have looked into the status of virus particles before and after sonication by electron microscopy (Figures 5A and 5B). Even in the absence of sonication, most virions exist as single, separate particles, with small fraction of virus particles in aggregates (not quantified). The virus particles display uniform size and shape. In addition, as expected, some cellular debris was visible. With sonication, all of the virions are in single particle, with some membranes disrupted (Figure 5B). The unsonicated and sonicated virus samples were titered on CV-1 cells and both showed the same titer, at 1.1e9 PFU/mL. These two experiments demonstrated that the virus preparations purified through our protocol, even after storing at a −80°C freezer for months, are mostly in single particles and there was no need to use sonication before the application of the virus for basic research or, presumably, for applications to human patients in the future.

**Figure 6:**
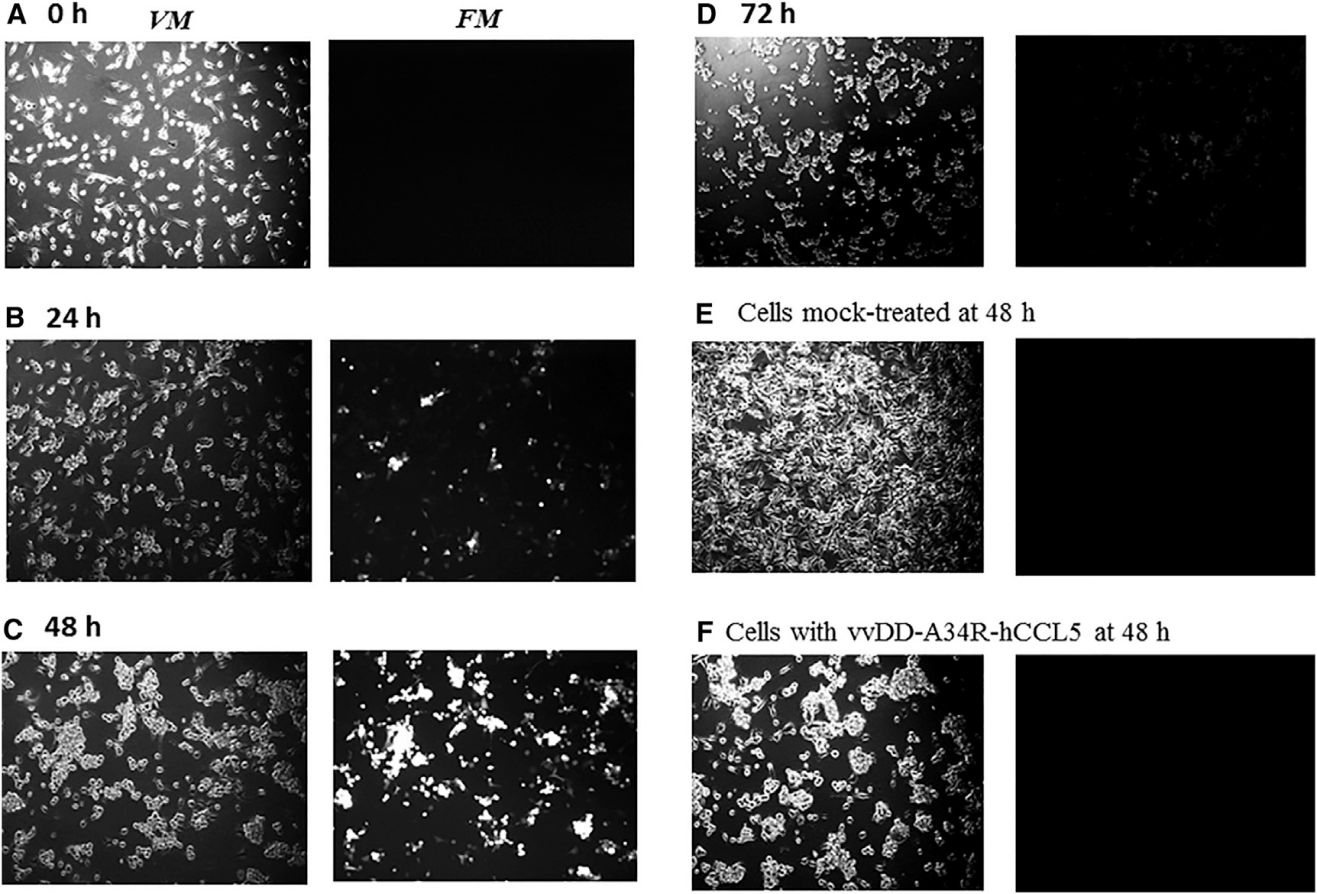
Viral Infection, Marker-Gene Expression, and Oncolysis in Infected MC38 Colon Cancer Cells Viewed through Microscopy MC38 cancer cells at 2.0e5 cells/well (in a 6-well culture plate) were infected with OVs vvDD-EGFP or vvDD-A34R-hCCL6 at MOI of 1.0. Shown are representative visible light (left; *VM*) and fluorescence (right; *FM*) microscopy of the MC38 cells infected with vvDD-EGFP at times 0 (A), 24 (B), 48 (C), and 72 hr (D). By 72 hr after infection, all cells were floating and dead. The last two panels showed the images of microscopy of MC38 cells mock-infected (E) or infected (F) with vvDD-A34R-hCCL5 at 48 hr.

We also demonstrated that vvDD-A34R-hCCL5 replicated efficiently in cancer cells, comparable to the parental virus vvDD in both MC38 and HCT116 cancer cells (Figures 5C and 5D). We have followed with the kinetics of viral infection, marker gene expression, and oncolysis in MC38 cancer cells (Figure 6). Using the virus expressing EGFP (vvDD-EGFP) to infect MC38 colon cancer cells at MOI of 1.0, it was demonstrated that the virus infects these cancer cells; by 24 hr, cells showed cytopathic effect (CPE), and ∼10%–30% of cells expressed EGFP (Figures 6A and 6B). This marker gene expression increases to 80%–100% by 48 hr after infection (Figure 6C). Essentially all MC38 cancer cells were detached, and little EGFP was expressed at this time. These floating cancer cells collected 72 hr post-infection were stained by trypan blue dye and examined by microscopy. They were mostly dead even though up to ∼30% were still alive by trypan blue dye exclusion assay (Figure 6E). Those infected with the clinical-grade virus (vvDD-A34R-hCCL5) showed the same kinetics of cell morphological changes as those infected with vvDD-EGFP (Figure 6F).

In summary, this improved procedure was efficient for the construction of recombinant poxviruses with reduced risk of adventitious mutations, with potential downstream application for both basic and clinical studies. Our study further validated the combination use of marker gene swapping and fluorescence-activated cell sorting established by Di Lullo,^21^ and the utility of the SEM system established by Rintoul et al.^42^ We have utilized this improved procedure several times for creating new recombinant VVs. The initial characterization of the novel oncolytic virus vvDD-A34R-hCCL5 showed the purification procedure for a clinical grade virus was also efficient. The virus behaves as a potent oncolytic virus in human and murine cancer cells and produces a high level of secreted CCL5 from infected human cells.

## Discussion

This study has two purposes. The first was to test a simplified procedure to generate high-fidelity recombinant poxviruses that meet the requirements of regulatory agencies for clinical applications, which requires a protocol that minimizes the mutation of the viral genome and removal of selectable markers. The second was to produce the new virus vvDD-A34R-hCCL5 in a clinical grade for planned clinical trial.

The study by Dambach et al.^35^ clearly documented the possibility of adventitious mutations in clinical grade viruses. A conclusion from that study is that an unmet need is a procedure for creating and selecting new recombinant viruses with minimizing cellular stress that might lead to unintended mutations across the viral genome. This avoids the potential need to sequence the entire recombinant virus genome following large-scale production for clinical translation.^35^, ^46^ Such an approach could cause additional delays and cost, especially for viral vectors with large genome sizes. There are a number of strategies to monitor the mutations of the viral vectors during the steps of the construction, before and after large-scale production. A good practice is to minimize unintended mutations from initial stages of vector construction and selection steps.

Di Lullo et al.^21^ have established a system in that cells infected by recombinant poxviruses can be obtained by fluorescence-activated cell sorting and new recombinant VV can be generated by a swapping event between different fluorescent protein genes in the acceptor virus and a plasmid cassette coding for the marker gene and a transgene. One of our groups has previously improved the system using the double selection and the SEM system.^42^ In the current study, we have successfully reproduced some main findings in these two previous studies. Using the improved procedure, we have been able to generate a number of recombinant VVs containing genetic manipulations in multiple genetic loci and with or without marker genes. Sequencing of manipulated DNA sequences in multiple loci in vvDD-A34R-hCCL5, the focus of this study, found no unintended mutations in the selected DNA segments of interest. These results on multiple viruses and multiple clones of each virus, albeit still relatively small in numbers, did validate our assumption that this procedure with low cellular stress was useful in generating new high-fidelity recombinant poxviruses for clinical studies.

It is also preferable to remove marker genes from recombinant viruses for multiple reasons. First and most importantly, a safe and antibiotic selection marker-free poxvirus vector is designed to comply with the regulatory guidance from both the US Food and Drug Administration and European Medicines Agency. Second, removal of a given marker allows reuse of the same marker for further genetic manipulation. Finally, marker genes and other inserted sequences in viral vectors may affect the overall fitness of the resulting virus, as shown in a number of cases.^47^, ^48^, ^49^, ^50^, ^51^, ^52^ Therefore, our study has confirmed the great advantages of the SEM system for the removal of marker genes.

In the middle of our study, Yuan et al.^53^ published a novel approach that utilized the CRISPR Cas9 system, in combination with Cre-loxp and Flp-FRET systems, to make marker-free VVs. Their new method appears to work very efficiently with the CRISPR Cas9 system used. The major difference between these two approaches is the step of generating recombinant VV: one by homologous recombination and the other through editing by the CRISPR Cas9 system. However, the CRISPR Cas9 system does have a weakness. It has well-documented off-target effects that will generate mutations at other genetic loci.^54^, ^55^ This high-frequency off-target mutagenesis may lead to multiple adventitious mutations throughout the viral genome and human genome in the host cells.^54^ In contrast, our method presented here of utilizing homologous recombination is highly reliable, and we needed only 1.0e5 CV-1 cells in a single well in a 6-well plate to obtain new recombinant VV. In summary, our approach provides a validated, highly reliable methodology for making marker-free and high-fidelity recombinant VVs for clinical applications.

In order to move forward to conduct a clinical trial with one of such constructed VV, vvDD-A34R-hCCL5, we have produced and purified a batch of this virus stock in clinical grade in the GMP facility. The production of VV for clinical study, just like other oncolytic viruses, is quite challenging as it needs to meet the strict regulatory requirements.^56^ We have performed a series of in vitro characterizations on the clinical-grade virus stock. The results of various tests showed that the virus preparation was sterile, did not contain adventitious agents, contained minimum content of endotoxin, and it existed mostly as single virion particles. We showed that this virus replicated as expected and induced oncolysis in multiple lines of cancer cells. It generated a comet plaque phenotype due to the production of many extracellular enveloped forms (EEVs) of the virus, a typical character with the specific A34R gene mutation.^45^ The virus produced and secreted a high level of chemokine CCL5 from infected cells. These studies indicated that this newly constructed VVs behaved as expected.

In summary, this low cellular stress procedure and the SEM system for removal of the marker genes should be very useful for construction of new marker-free, high-fidelity recombinant VVs intended for human clinical studies as well as basic studies. We have prepared a batch of the virus vvDD-A34R-hCCL5 in clinical grade that appears to meet the parameters for phase I studies in the in vitro characterizations.

## Materials and Methods

### Cell Lines

CV-1, HEK293, and HeLa cell lines were originally obtained from ATCC (Manassas, VA). The Cre recombinase-expressing human osteosarcoma U2OS-Cre cells were created in the previous study.^42^ Other cancer cell lines, such as MC38 murine colon cancer and HCT116 human colorectal cancer, have been used in our previous studies. These cells were grown in DMEM medium supplemented with 5%–10% heat-inactivated fetal bovine serum (FBS; Atlanta Biologicals, Flowery Branch, GA), 100 U/mL penicillin and 100 ng/mL streptomycin (Gemini Bio-Products, Sacramento, CA). Culture cells have been monitored to ensure that they are mycoplasma-free using MycoAlert Plus Mycoplasma Detection Kit (Lonza, Rockland, ME).

### Recombinant VVs

Western Reserve (WR) strain VVs were obtained originally from Dr. Bernard Moss at the National Institute of Allergy and Infectious Diseases, NIH. This WR strain of VV has been used in our studies since 1999. The genetic manipulations in the virus have targeted three genetic loci: genes encoding thymidine kinase (*tk*) (gene name: J2R or VACWR094), vaccinia growth factor (*vgf*) (C11R or VACWR009), and A34R (VACWR157). Recombinant viruses vSC20 (*vgf* gene mutated) and vvDD (*J2R* and *C11R* mutations) have been described previously.^44^ The parental virus used for new construction is vSC20. In order to create new viruses by homologous recombination in CV-1 cells, new shuttle plasmids for the *tk*, *vgf*, and A34R gene loci have been constructed. Some representative plasmids are described in Figure 1. To generate new VVs, CV-1 cells in one well of a 6-well plate were infected with a parental virus at MOI of 0.1 for 2 hr, then transfected with 2 μg of the shuttle vector using TurboFect transfection reagent (Thermo Fisher Scientific, Waltham, MA). Two days later, cells and supernatant were harvested for flow cytometry or other analyses.

### The Initial Steps in Creating New Recombinant VV

To create vvDD-hCCL5-YFP, CV-1 cells in a 6-well plate (∼80% confluency) were infected with vSC20 at MOI of 0.1 for 2 hr, then transfected with plasmid pMS-hCCL5 using TurboFect transfection reagent (Thermo Fisher Scientific). Two days later, we observed about 15%–30% YPF-positive cells by visible light and fluorescence microscopy. Cells and supernatants were harvested, and the lysates were used to infect new CV-1 cells in one well of a 6-well plate. All cells were harvested around 24 hr later and subjected to flow sorting, using uninfected cells or cells infected with a well-characterized YFP-expressing VV as negative and positive controls for gating purposes. The YFP-positive cells were sorted into CV-1 cells in 96-well plates, one cell per well for one (or two) 96-well plates per new virus (Figure 2, steps 1 and 2).

### Flow Sorting

CV-1 cells were infected with new recombinant and parental virus mixture. At 20–24 hr post infection, cells were harvested and subject to single-cell sorting. Single YFP-positive cells were sorted at one cell per well into CV-1 cells cultured in 96-well plates. For each new virus, we normally sorted YFP-positive cells into wells of CV-1 cells (50%–80% confluent) in 96-well plates, at one positive cell per well. In most cases, one 96-well plate of CV-1 cells was used for one new virus.

Cell sorting was performed with MoFlo Astrio EQ Flow Cytometry System (Beckman Coulter, Brea, CA). Technical assistance was provided by the Flow Cytometry Core Facility located at UPMC Hillman Cancer Center.

### Visible Light and Fluorescence Microscopy

The state of cells and expression of fluorescent proteins (YFP and eGFP) from infected cells were visualized and electronically recorded with an inverted microscope (Zeiss) that was linked to a computer with the software AxioVision (release 4.8.1) for image documentation from Carl Zeiss Microscopy (Peabody, MA).

### Cloning and DNA Sequencing

Restriction enzymes were ordered from New England Biolabs (Ipswich, MA). Rapid DNA ligation kit was from Roche Diagnostics (Indianapolis, IN). Primers for PCR and sequencing were designed with the help of Primer3 online software, and primers were ordered from Integrated DNA Technologies (Coralville, IA). The primers for sequencing are listed in Table 2.

**Table 2:** Primers Used for DNA Sequencing

	Sequences (5′–3′)
LP12	CGCATTTTCTAACGTGATGG
RP1427	AACACTTTCTACACACCGATTGA
LP84	GAACGGCGGACATATTCAGT
RP1372	GGTTTCCTCACCCAATCGTT
LP84	GAACGGCGGACATATTCAGT
LP469	CCCCCTGAACCTGAAACATA
LP659	CTCAAGTGATCCACCCACCT
LP939	ACACACTTGGCGGTTCTTTC
LP1220	ATAGTAGCCGCACTCGATGG
RP271	TTGCTTCCAATGCTTCAAAA
RP678	AGGTGGGTGGATCACTTGAG
RP813	GAGGCTTCCCCTCACTATCC
RP1134	CGCTGTCATCCTCATTGCTA
RP1204	GTTTGCCATACGCTCACAG
LP-A34R121	GAACTGATGCCTAGTGCTTG
RP-A35R875	CCATTGCCGTCTGATATGC
LP-A34R399	CAGTACGACGGATGCTGAAG

Viral genomic DNA was purified using a DNeasy Blood and Tissue Kit (QIAGEN, Valencia, CA) according to manufacturer’s instructions. PCR was conducted with 30 ng viral genomic DNA, Taq 2x Master Mix (New England Biolabs), through 35 cycle of amplification. PCR products were purified using High Pure PCR Product Purification Kit (Roche Diagnostics). DNA sequencing was performed by the University of Pittsburgh Genomics Research Core Facility.

### Characterization of the Viruses In Vitro

Cancer cells were seeded at 2.0e5 cells per well in 6-well plates, and next day they were mock-infected or infected with either vvDD-EGFP or vvDD-A34R-hCCL5 at MOI of 1.0 and were harvested at 12, 24, 36, 48, and 72 hr after infection. The yields of the virus were titered in CV-1 cells by plaque assays and expressed as yield of total PFU. At various time points, cells were monitored for GFP expression and cytotoxicity by visible light and fluorescence microscopy.

### ELISA Assay for Human and Murine CCL5

Human HEK293 cells were plated at 1.0e5 per well in 6-well plates. At 24 hr after seeding, cells were mock-infected or infected with vvDD-EGFP or vvDD-A34R-hCCL5 at MOI of 1.0. The conditioned media were collected at 24 and 48 hr post-infection. The concentration of human CCL5 was quantified using a Quantikine ELISA kit (cat. no. DRN00B; R&D Systems; Minneapolis, MN).

### Production of Clinical-Grade vvDD-A34R-hCCL5

The production of the clinical-grade virus was performed in the UPCI Immunological Monitoring and Cellular Products Laboratory (IMCPL). The laboratory is CAP inspected, CLIA certified, FACT accredited, and maintains a Facilities Master File with the FDA. The manufacturing process was performed according to many but not all aspects of Good Manufacturing Practice (GMP) to meet the requirements of product testing for phase I clinical trial setting.

The infection of HEK293 cells and purification of the virus from cell lysate are described briefly as follows. When HEK293 cells in the Cell Factories were just about to be confluent, the media were removed. The 300 mL of DMEM with 2% FBS and the virus lysate (seed stock) was added through sterile funnel. The infection lasted for 2 hr with the apparatus gently swirled once every 20 min. Then 700 mL of complete growth medium (DMEM + 10% FBS + pen/strep) was added to each Cell Factory using a sterile funnel, and the Cell Factories were returned to an incubator (37°C, 5% CO_2_). The state of infection was monitored over time using a microscope.

Fifty hours after infection, infected cells, media, and released virus were collected from the Cell Factories. Each Cell Factory was washed with 500 mL 1× PBS total and collected and pooled. Pooled cells were centrifuged for 5 min at 2,000 rpm and resuspended in 14 mL 10 mM Tris-HCl (pH 9.0). The cells were homogenized and spun for 10 min at 300 × *g*. The supernatant was sonicated using a cup sonicator. Then the lysate was adjusted to final concentrations of 50 U/mL of benzonase nuclease, 2 mM MgCl2, and 5 mM Tris-HCl (pH 9.0) and incubated on a shaker for 2 hr at room temperature. The lysate was further treated with 1× TrypLE (Thermo Fisher Scientific) for 30 min with shaking at room temperature.

The enzyme-treated and cleared cell lysate was layered onto 36% sucrose solution in centrifuge tubes and spun for 1 hr at 16,000 rpm and 4°C in a SW28 rotor. The pellets were resuspended in 10 mM Tris-HCl (pH 9.0) and layered over 24%–40% continuous sucrose gradient in sterile SW28 centrifuge tubes (which were prepared 1 day before) and spun for 50 min at 12,000 rpm and 4°C. The virus band as milky band in the middle of the tube as well as pellet were collected, resuspended and pooled, and pelleted again. The pellets were resuspended in 10 mM Tris-HCl buffer (pH 9.0). The virus suspension was aliquoted into 250 μL in vials.

## Author Contributions

Z.S.G., Z.L., and M.S. designed and performed most of the experiments and analyzed data. R.R. helped with some experiments. J.W. and J.C.B. provided plasmids, key cell lines, and technical advice. E.K., S.H., T.W.K., L.H.B., and A.G. were responsible for the production of the clinical grade virus. H.J.Z. and D.L.B. provided conceptual advice. Z.S.G. prepared the manuscript.

## Conflicts of Interest

Z.S.G. is a scientific advisor to ICell Kealex Therapeutics and J.W., J.C.B., and D.L.B. have financial interests with SillaJen Biotherapeutics, biotech companies developing oncolytic viruses. The other authors declare no conflict of interest.
